# Prevalence and Risk Factors of Gastroesophageal Reflux Disease in Southwestern Saudi Arabia

**DOI:** 10.7759/cureus.6626

**Published:** 2020-01-10

**Authors:** Ali M Kariri, Mohammad A Darraj, Almonther Wassly, Hassan A Arishi, Majid Lughbi, Alhassan Kariri, Abdullah M Madkhali, Musab I Ezzi, Basim Khawaji

**Affiliations:** 1 Internal Medicine, Jazan University, Jazan, SAU

**Keywords:** gerd, reflux, risk factors, prevalence, saudi arabia

## Abstract

Background

Gastroesophageal reflux disease (GERD) is one of the most common gastrointestinal disorders that has substantial health and economic consequences. Several modifiable risk factors are associated with GERD, hence we conducted the present study to assess the prevalence and risk factors of GERD in a previously understudied population of southwestern Saudi Arabia.

Methods

A cross-sectional study was carried out to calculate the prevalence of GERD and assess its risk factors. A structured self-administered questionnaire was distributed on a random sample of 853 participants from Jazan region, Saudi Arabia. The questionnaire consisted of questions on the participants’ sociodemographic and lifestyle characteristics. The presence of GERD was detected using the GERD questionnaire (GerdQ). Data were analyzed using descriptive statistics and chi-square test, with a significance level of P < 0.05 or P < 0.01.

Results

The study included 853 participants; 69.1% males and 30.9% females. The proportion of participants who scored >8 on the GerdQ (had GERD) was 32.2%. GERD was associated with age (P < 0.01), marital status (P < 0.01), employment status (P < 0.01), fast food intake (P < 0.01), analgesics use (P < 0.01), and smoking (P < 0.01). GERD was more common among Khat chewers compared to non-Khat chewers (P < 0.05) and showed a significant association with the frequency of Khat use.

Conclusion

The results show a high prevalence of GERD in the general population of Southwestern Saudi Arabia. Several sociodemographic and lifestyle characteristics were associated with the disease.

## Introduction

Some degree of retrograde flow of stomach contents to the esophagus is physiologic. Gastroesophageal reflux disease (GERD) develops when these retrograde flows cause troublesome acid heartburn and/or regurgitation at least one day a week [1]. GERD is well-known for its high prevalence, variety of symptoms, and its impact on the patients’ quality of life and productivity. Also, GERD has substantial economic consequences [2].

Prior research showed that GERD affected 10% to 20% of individuals in the Western world, with a comparatively low prevalence (5%) among Asians [3]. Studies from Saudi Arabia reported a range of GERD prevalence of 23.5% and 45.4% [4-6]. However, endoscopy-based studies found lower estimates of the prevalence of GERD [7,8].

Several lifestyle-related and modifiable risk factors for GERD have been demonstrated. GERD was found to be more common in individuals who used non-steroidal anti-inflammatory drugs (NSAIDs) and individuals who were smoker, obese, and physically inactive [9-12]. Certain types of food and drinks, such as fast food, coffee, tea, greasy food, and carbonated drinks have been linked to increased prevalence of GERD [13,14]. One Ethiopian study suggested that Khat (a psycho-stimulant substance commonly used in Ethiopia, Yemen, Madagascar, South Africa, Kenya, Sudan S, and Southwestern Saudi Arabia) may be a predisposing factor to gastrointestinal disorders including GERD [15].

Data on the prevalence and risk factors for GERD in Southwestern Saudi Arabia are scarce. Therefore, we conducted this survey aiming to assess the prevalence and risk factors for GERD in the general population of Jazan region, Saudi Arabia.

## Materials and methods

A descriptive cross-sectional study was conducted between July 2019 and October 2019 in Jazan (also pronounced Gizan) region, Southwestern Saudi Arabia. The total Saudi population in Jazan is 1,228,553 individuals at the 2018 census [16]. Therefore, the minimum required sample size was 385 with a confidence level of 95% and a confidence interval (CI) 5%. With a confidence level of 99% and a CI of 5%, a sample of 664 was calculated and randomly collected. Accounting for a non-response rate of 25%, the final sample size was 830 participants. Twenty-three more questionnaires were collected as some subjects asked to volunteer after the required sample was reached. A total of 345 (40.6%) questionnaires were collected using an online link to a self-administered questionnaire. The remaining 505 (59.4%) questionnaires were collected by the study authors in different primary care centers, two large malls, and Jazan University. The study included all Saudi general population in Jazan region who were over 18 years of age and gave informed consent to take part in the study. All participants were informed of the study objectives and the anonymity of the questionnaire. The study was approved by Jazan University’s institutional review board.

Since there is no validated Arabic version of the GERD questionnaire (GerdQ), the study authors translated the English version into simple Arabic using back-translation, which was approved by two independent internal medicine consultants. The questionnaire consisted of questions on the subjects’ baseline characteristics (sex, age, marital status, employment status, education, and height and weight) and lifestyle characteristics (smoking, Khat chewing, and types of food and drinks). Given the high prevalence of Khat chewing in Jazan Province, detailed history of the frequency of Khat chewing was obtained and categorized ascendingly into not at all, occasionally (only in holydays), monthly, weekly, alternating days, and daily [17]. The body mass index (BMI) was calculated using the following formula: weight (kg) / [height (m)]^2^.

The GerdQ is a six-item diagnostic tool that helps diagnose and monitor GERD symptoms [18]. The six items of GerdQ assess the frequency of four positive GERD predictors (regurgitation, heartburn, sleep disturbance due to heartburn and/or regurgitation, and the use of over the counter (OTC) medications other than the typical GERD medications). Each symptom is rated on a 4-point Likert scale (0 = 0 day, 1 = 1 day, 2 = 2-3 days, and 3 = 4-7 days). The negative GERD predictors (nausea and epigastric pain) are rated reversely (3 = 0 day). The total score was calculated and a diagnosis of GERD was made using a cut-off score of >8 [18]. The Cronbach’s alpha for the GerdQ was 0.87, indicating good internal consistency. GerdQ has a specificity and sensitivity of 71% and 65%, respectively [18].

The statistical analysis included descriptive statistics presented as well as Chi-square test and for comparison between groups. Differences were considered significant if P <0.05 or <0.01. Data was analyzed using the Statistical Package of Social Sciences (SPSS) Version 20 (IBM Corp., Armonk, NY).

## Results

Baseline characteristics of the study population

The study included 853 participants, with 69.1% males and 30.9% females. Over a half (58.9%) of the participants were aged 36-50 years and 38.5% were married. Over three-quarters (75.6%) had a college degree or higher education and 37.4% were employed. Participants with obesity represented 16.4% of the sample. As per the GerdQ criteria, 32.2% of the study population had GERD (scored >8). A diagnosis of GERD was significantly associated with age group, marital status, and employment status (all P-values <0.01). Socio-demographic characteristics of participants with and without GERD are detailed in Table 1.

**Table 1:** Baseline characteristics of the study population GERD: Gastroesophageal reflux disease.

Characteristics	Total (N = 853)		GERD %		P
	N	%	No (N = 578)	Yes (N = 275)	
Age (years)					
19-25	450	52.8	76.9	23.1	0.000
26-35	198	23.2	64.6	35.4	
36-50	124	14.5	41.1	58.9	
50 or older	81	9.5	65.4	34.6	
Gender					
Male	589	69.1	69.8	30.2	0.068
Female	264	30.9	63.3	36.7	
Marital status	182	100.0			
Not married	525	61.5	73.3	26.7	0.000
Married	328	38.5	58.8	41.2	
Education	37	20.3			
Elementary or intermediate school	30	3.5	50.0	50.0	0.089
Secondary school	178	20.9	70.2	29.8	
College degree or higher	645	75.6	67.9	32.1	
Employment status	182	100.0			
Not employed	139	16.3	64.7	35.3	0.000
Student	355	41.6	76.1	23.9	
Employed	319	37.4	60.5	39.5	
Retired	40	4.7	62.5	37.5	
Body mass index (BMI)					
Underweight	61	7.2	73.8	26.2	0.396
Normal weight	304	35.6	70.1	29.9	
Overweight	247	29.0	66.4	33.6	
Class I obesity	140	16.4	64.7	35.3	

Lifestyle characteristics of the study population

**Figure 1:**
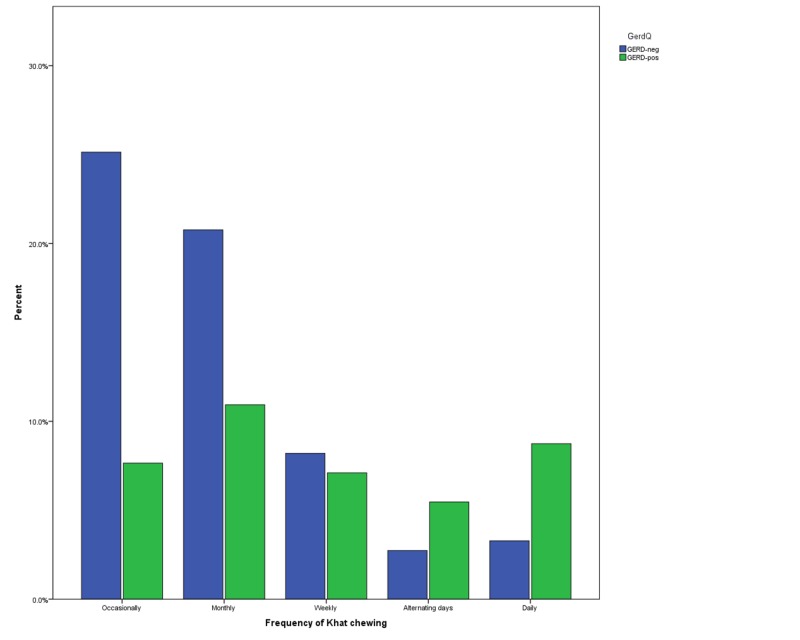
Khat chewing frequency and GERD GERD: Gastroesophageal reflux disease

As shown in Table 2, the most common type of food consumed by the study populations was fast food (38.1%) and the most common type of drinks was tea/coffee (34.6%). A total of 31.3% used NSAIDS regularly. Smoking was self-reported by one-third of the participants (33.3%) and Khat chewing was practiced by 21.6%. GERD was significantly associated with all lifestyle characteristics shown in Table 2 (all P <0.01 or 0.05). A detailed description of the relationship between GERD and frequency of Khat chewing is illustrated in Figure 1.

**Table 2:** Lifestyle characteristics of the study population GERD: Gastroesophageal reflux disease; NSAIDs: Non-steroidal anti-inflammatory drugs.

Characteristics	Total (N = 853)		GERD %		P
	N	%	No (N = 578)	Yes (N = 275)	
Eating and drinking habits					
Fast food	325	38.1	57.5	42.5	0.000
Spicy food	81	9.5	65.4	34.6	
Soft drinks	152	17.8	67.1	32.9	
Tea/coffee	295	34.6	80.0	20.0	
Analgesic use (NSAIDs)					
No	586	68.7	72.4	27.6	0.000
Yes	267	31.3	57.7	42.3	
Smoking					
No	569	66.7	71.7	28.3	0.001
Yes	284	33.3	59.9	40.1	
Khat chewing frequency					
No	669	78.4	69.8	30.2	0.016
Yes	184	21.6	60.3	39.7	

## Discussion

This cross-sectional study demonstrated a high prevalence (32.2%) of GERD symptoms in the general population of southwestern Saudi Arabia. GERD was associated with many sociodemographic and lifestyle characteristics. In addition, we found a unique relationship between the presence of GERD and Khat chewing, a relationship that has been investigated by few studies in the literature. Previous research has shown that GERD was common in the general population of Saudi Arabia, with variable estimated prevalence rates possibly attributed to different diagnostic tools used (questionnaire-based vs. endoscopy-based) and different populations investigated. In two studies conducted in western and central Saudi Arabia, GERD affected 23.5% and 45.4%, respectively [5,6]. Alsuwat et al. surveyed 2,043 participants from the 13 administrative regions of Saudi Arabia and reported a prevalence rate of 28.7% [4]. Like the present study, these studies used the GerdQ with a cut-off point of >8 to indicate GERD presence. Al-Humayed et al. performed upper gastrointestinal endoscopy on 1,607 patients from the southern region of Saudi Arabia and found 15% of them to have GERD [8].

In agreement with previous studies, we found an association between GERD and age [19,20]. Although females in our study (36.7%) suffered GERD symptoms more than did males (30.2%), the relationship between GERD and gender was not of statistical significance. These findings are supported by what has been reported by previous studies [4]. Regarding marital status, the analysis showed that married people were more likely to have GERD symptoms than single people (including divorced and widows). This relationship between GERD and marital status has been reported by other authors [21]. Higher education was associated with a lower prevalence of GERD in some of the previous studies [22], though other studies found no link between education and GERD [4]. We demonstrated no difference in GERD prevalence with different education levels. One possible explanation is that most of the studied population are highly educated, reflecting a non-normal distribution of education in the current sample. Employment status was associated with GERD in this study, which is consistent with what has been reported by other studies [23].

The analysis showed that GERD was significantly related to lifestyle factors. We found that GERD was more common among people who regularly consumed fast food, used analgesics (NSAIDs), and those who were regular smokers. These findings have been demonstrated by other studies. A detailed discussion of these associations can be found in the study of Alkhathami et al. [13]. Previous research has shown that the prevalence of GERD was associated with certain types of food and drinks, the use of analgesics, sleep habits, and smoking [24-27].

Khat (Catha edulis Forsk) is a plant with psycho-stimulant properties that commonly cultivated in Ethiopia, Yemen, Madagascar, South Africa, Kenya, and Sudan. Khat chewers use the fresh leaves and buds of Khat for psychological and social reasons [28]. Although gastroenterological adverse effects of Khat have been reported, only a few studies investigated Khat as a precipitating factor of GERD [15]. We observed that GERD symptoms were more common in Khat chewers compared with non-Khat chewers, as well as in heavy chewers (defined as daily consumption of Khat) and frequent chewers compared to occasional chewers. Figure 1 depicts the relationship between Khat chewing frequency and GERD, which shows that the presence of GERD symptoms increased proportionally with Khat chewing frequency (Figure 1). Nigussie et al. found similar results in a sample of 1005 Ethiopian university students. In their study, gastrointestinal disorders, including GERD, constipation, gastritis, and hemorrhoids, were more common among Khat chewers than non-chewers [15]. These findings should motivate future research to further explore the role of Khat use in GERD and to find out whether it is explainable by factors intrinsic to the Khat itself or lifestyle factors that are unique to Khat chewers or a combination of both factors. The relatively large sample size and different methods of data collection add to the strength of the study and allows the generalizability of its findings. However, recall bias, which is common in such studies, and the use of GerdQ, which has not yet been validated in the studied population, are important limitations. Nonetheless, our findings pointed out the high prevalence of GERD and its association with sociodemographic and lifestyle characteristics, which need to be validated by further research.

## Conclusions

This study revealed that GERD is common in the general population of the southwestern region of Saudi Arabia. GERD was associated with the sociodemographic and lifestyle characteristics of the studied population. Sociodemographic factors that are associated with GERD included age, marital status, and employment status. Lifestyle factors are fast food intake, analgesics use, smoking, and Khat chewing.

## References

[1] Vakil N, van Zanten SV, Kahrilas P, Dent J, Jones R; Global Consensus Group (2006). The Montreal definition and classification of gastroesophageal reflux disease: a global evidence-based consensus. Am J Gastroenterol.

[2] DeVault KR, Castell DO; American College of Gastroenterology (2005). Updated guidelines for the diagnosis and treatment of gastroesophageal reflux disease. Am J Gastroenterol.

[3] Dent J, El-Serag HB, Wallander M-A, Johansson S (2005). Epidemiology of gastro-oesophageal reflux disease: a systematic review. Gut.

[4] Alsuwat OB, Alzahrani AA, Alzhrani MA, Alkhathami AM, Mahfouz MEM (2018). Prevalence of gastroesophageal reflux disease in Saudi Arabia. J Clin Med Res.

[5] Almadi MA, Almousa MA, Althwainy AF (2014). Prevalence of symptoms of gastroesopahgeal reflux in a cohort of Saudi Arabians: a study of 1265 subjects. Saudi J Gastroenterol.

[6] Binhussein M, Alamoudi A, Bajawi A (2016). Prevalence of gastroesophageal reflux in western region of Saudi Arabia. Saudi J Gastroenterol.

[7] Zagari RM, Fuccio L, Wallander M-A (2008). Gastro-oesophageal reflux symptoms, oesophagitis and Barrett’s oesophagus in the general population: the Loiano-Monghidoro study. Gut.

[8] Al-Humayed SM, Mohamed-Elbagir AK, Al-Wabel AA, Argobi YA (2010). The changing pattern of upper gastro-intestinal lesions in southern Saudi Arabia: an endoscopic study. Saudi J Gastroenterol.

[9] Mahadeva S, Raman MC, Ford AC, Follows M, Axon AT, Goh KL, Moayyedi P (2005). Gastro-oesophageal reflux is more prevalent in Western dyspeptics: a prospective comparison of British and South-East Asian patients with dyspepsia. Aliment Pharmacol Ther.

[10] Saberi-Firoozi M, Khademolhosseini F, Yousefi M, Mehrabani D, Zare N, Heydari ST (2007). Risk factors of gastroesophageal reflux disease in Shiraz, southern Iran. World J Gastroenterol.

[11] Wong WM, Lam KF, Lai KC (2003). A validated symptoms questionnaire (Chinese GERDQ) for the diagnosis of gastro-oesophageal reflux disease in the Chinese population. Aliment Pharmacol Ther.

[12] Jarosz M, Taraszewska A (2014). Risk factors for gastroesophageal reflux disease: the role of diet. Prz Gastroenterol.

[13] Alkhathami AM, Alzahrani AA, Alzhrani MA, Alsuwat OB, Mahfouz MEM (2017). Risk factors for gastroesophageal reflux disease in Saudi Arabia. Gastroenterol Res.

[14] Arivan R, Deepanjali S (2018). Prevalence and risk factors of gastro-esophageal reflux disease among undergraduate medical students from a southern Indian medical school: a cross-sectional study. BMC Res Notes.

[15] Nigussie T, Gobena T, Mossie A (2013). Association between khat chewing and gastrointestinal disorders: a cross sectional study. Ethiop J Health Sci.

[16] (2019). General Authority for Statistics. Population characteristics surveys. Population Characteristics surveys.

[17] Mahfouz MS, Rahim BEA, Solan YMH, Makeen AM, Alsanosy RM (2015). Khat chewing habits in the population of the Jazan region, Saudi Arabia: prevalence and associated factors. PLoS ONE.

[18] Jones R, Junghard O, Dent J, Vakil N, Halling K, Wernersson B, Lind T (2009). Development of the GerdQ, a tool for the diagnosis and management of gastro-oesophageal reflux disease in primary care. Aliment Pharmacol Ther.

[19] He J, Ma X, Zhao Y (2010). A population-based survey of the epidemiology of symptom-defined gastroesophageal reflux disease: the Systematic Investigation of Gastrointestinal Diseases in China. BMC Gastroenterol.

[20] Mohammed I, Cherkas LF, Riley SA, Spector TD, Trudgill NJ (2003). Genetic influences in gastro-oesophageal reflux disease: a twin study. Gut.

[21] Pourhoseingholi A, Pourhoseingholi MA, Moghimi-Dehkordi B (2012). Epidemiological features of gastro-esophageal reflux disease in Iran based on general population. Gastroenterol Hepatol Bed Bench.

[22] Wang H-Y, Leena KB, Plymoth A, Hergens MP, Yin L, Shenoy KT, Ye W (2016). Prevalence of gastro-esophageal reflux disease and its risk factors in a community-based population in southern India. BMC Gastroenterol.

[23] Niu C-Y, Zhou Y-L, Yan R, Mu NL, Gao BH, Wu FX, Luo JY (2012). Incidence of gastroesophageal reflux disease in Uygur and Han Chinese adults in Urumqi. World J Gastroenterol.

[24] Ebrahimi-Mameghani M, Sabour S, Khoshbaten M, Arefhosseini SR, Saghafi-Asl M (2017). Total diet, individual meals, and their association with gastroesophageal reflux disease. Heal Promot Perspect.

[25] Eusebi LH, Ratnakumaran R, Yuan Y, Solaymani-Dodaran M, Bazzoli F, Ford AC (2018). Global prevalence of, and risk factors for, gastro-oesophageal reflux symptoms: a meta-analysis. Gut.

[26] Jung H-K, Choung RS, Talley NJ (2010). Gastroesophageal reflux disease and sleep disorders: evidence for a causal link and therapeutic implications. J Neurogastroenterol Motil.

[27] Nocon M, Labenz J, Willich SN (2006). Lifestyle factors and symptoms of gastro-oesophageal reflux -- a population-based study. Aliment Pharmacol Ther.

[28] Al-Motarreb A, Baker K, Broadley KJ (2002). Khat: pharmacological and medical aspects and its social use in Yemen. Phytother Res.

